# Odontogenic Keratocysts Don't Harbor BRAF Mutation: A Genetic and Immunohistochemical Analysis

**DOI:** 10.1111/odi.70358

**Published:** 2026-05-05

**Authors:** Raisa Severino‐Lazo, Jéssica da Silva Cunha, Allan Vinícius Martins‐de‐Barros, Karina Helen Martins, Adauto Gomes Barbosa Neto, Jorge Esquiche León, Marianne de Vasconcelos Carvalho

**Affiliations:** ^1^ University of Pernambuco, Oral HistoPathology Research Group, Graduate Program in Dentistry, Faculty of Dentistry of Pernambuco (FOP/UPE) University of Pernambuco Recife Pernambuco Brazil; ^2^ Programa de Pós‐Graduação Em Saúde e Desenvolvimento Socioambiental, Campus Arcoverde University of Pernambuco Arcoverde Pernambuco Brazil; ^3^ Programa de Pós‐Graduação Em Ciências da Saúde, Campus Santo Amaro University of Pernambuco Recife Pernambuco Brazil; ^4^ Department of Pediatric Dentistry, Ribeirão Preto Dental School University of São Paulo (FORP/USP) Ribeirão Preto Brazil; ^5^ University of Pernambuco. Laboratório Multiusuário Em Saúde Institute of Biological Sciences Recife Pernambuco Brazil; ^6^ Department of Pathology and Forensic Medicine, Ribeirão Preto Medical School (FMRP/USP) University of São Paulo Ribeirão Preto Brazil; ^7^ Oral Pathology, Department of Stomatology, Public Oral Health, and Forensic Dentistry, Ribeirão Preto Dental School (FORP/USP) University of São Paulo Ribeirão Preto Brazil; ^8^ University of Pernambuco (UPE), integrated Center of Pathological Anatomy Oswaldo Cruz University Hospital Recife Brazil

**Keywords:** BRAF V600E, odontogenic cyst, odontogenic keratocyst, proto‐oncogene proteins B‐raf

Odontogenic keratocyst (OKC) is a developmental cystic lesion arising from odontogenic epithelium remnants, most often in the posterior mandible of young adults with male predominance (World Health Organization Classification of Tumours Editorial Board 2023). Despite being benign, OKC shows aggressive behavior, including bone destruction and recurrence rates of 5%–62% depending on treatment (Gomes et al. 2009; Liu et al. 2025). Histologically, OKC displays parakeratinized stratified squamous epithelium and frequent *PTCH1* mutation in the Sonic Hedgehog (SHH) pathway (Gomes et al. 2017), supporting its earlier classification as a cyst with neoplastic potential and highlighting the need to clarify its molecular drivers (World Health Organization Classification of Tumours Editorial Board 2023; Liu et al. 2025).

The *BRAF* gene encodes a serine–threonine kinase in the MAPK pathway, regulating cell proliferation and survival (Duarte‐Andrade et al. 2022). The *BRAF* p.V600E missense mutation constitutively activates MAPK and is a recognized oncogenic driver in different types of cancer (Gomes 2022; Estrella et al. 2015). In ameloblastoma, *BRAF* p.V600E is consistently detected, although recent survival and meta‐analytic studies by our group show no reliable correlation with growth or recurrence, despite its relevance for targeted therapy development (Zhang et al. 2024; Martins‐de‐Barros et al. 2022; Severino‐Lazo et al. 2023). Its presence in OKC remains uncertain; although molecular overlap is suggested, most studies report negative findings (França et al. 2018; Cunha, J. da, et al. 2025).

Detection of *BRAF* p.V600E relies on immunohistochemical (IHC) or molecular assays, each with advantages and limitations. IHC using VE1 or RM8 monoclonal antibodies visualizes mutant protein in situ, providing rapid and cost‐effective screening (Martins‐de‐Barros et al. 2022; Krishnamurthy et al. 2018). In ameloblastomas, Martins‐de‐Barros et al. (2022) reported high sensitivity but lower specificity for RM8, largely due to false‐positive staining, reinforcing the need for molecular confirmation. Fisher et al. (2014) and Krishnamurthy et al. (2018) similarly noted that nonspecific cytoplasmic reactivity may mimic true mutation‐related staining, especially in keratin‐rich or suboptimally preserved tissue (Krishnamurthy et al. 2018; Fisher et al. 2014). Molecular techniques remain the gold standard for detecting single nucleotide substitutions (Gomes 2022; Estrella et al. 2015), though they require well‐preserved DNA and specialized infrastructure (Diniz et al. 2017). Given conflicting literature, this study aimed to evaluate the *BRAF* p.V600E in OKC using IHC and real‐time PCR (qPCR) to clarify its molecular status and pathogenic relevance.

This study was approved by the Institutional Review Board of the University of Pernambuco (Protocol No. 4.309.512). Twenty‐eight OKCs were analyzed, including two syndromic lesions associated with nevoid basal cell carcinoma syndrome. Case data were reviewed, and OKC diagnoses were confirmed by two oral pathologists. Formalin‐Fixed Paraffin‐Embedded (FFPE) samples were tested for *BRAF* p.V600E expression by IHC, using RM8 on full sections and VE1 on tissue microarray (TMA) sections. Cytoplasmic staining in ≥ 20% of epithelial cells was considered positive (Krishnamurthy et al. 2018; Fisher et al. 2014). IHC evaluation was independently performed by two experienced oral pathologists, both blinded to the molecular results, and any discrepant cases were resolved by consensus. All samples were analyzed by qPCR for molecular confirmation. Molecularly confirmed *BRAF* p.V600E–mutated ameloblastomas were used as positive controls for RM8 and as control tissue for VE1 staining; negative controls were obtained by omission of the primary antibody.

Genomic DNA was isolated from FFPE samples using the MagMax FFPE DNA/RNA Ultra Kit (Applied Biosystems, Thermo Fisher Scientific), quantified with a NanoDrop spectrophotometer, and mutation detection using TaqMan castPCR assays specific for the *BRAF* c.1799 T>A substitution (50 ng DNA input). Molecularly confirmed *BRAF* p.V600E–mutated ameloblastomas were included as positive controls in each qPCR run (Estrella et al. 2015; Martins‐de‐Barros et al. 2022).

The mean patient age was 35.92 years for sporadic cases, and 19 years for syndromic cases. Most lesions were mandibular (96.1%), with a slight male predominance (65.4%) among the sporadic group. The syndromic cases included one male and one female (Table 1). The qPCR revealed wild‐type *BRAF* in all samples. By IHC, RM8 showed one positive and one negative syndromic case, whereas VE1 was positive. Among sporadic OKCs, RM8 revealed 9 positive cases (34.61%), 14 negatives (53.85%), and 3 untested (11.54%). Using VE1, 16 sporadic cases (61.54%) were positive, 3 negative (11.54%), 3 untested (11.54%), and 4 had insufficient material (Figure 1).

**TABLE 1:** Main clinical features of OKC.

Continuous variables	Sporadic (*n* = 26)			Syndromic (*n* = 2)		
	Mean	SD	Range	Mean	SD	Range
Age at diagnosis (years)	35.92	18.48	10–80	19	2,83	21–17
Tumor size (cm^3^)	3.91	5.14	0.12–21.0	1.04	1.20	0.04–2.75
Categorical variables	**Number**		**Percentage**	**Number**		**Percentage**
Sex						
Male	17		65.4%	1		50%
Female	9		34.6%	1		50%
Location						
Mandible	25		96.1%	—		—
Maxilla	1		3.9%	—		—
Both				2		100%

**FIGURE 1:**
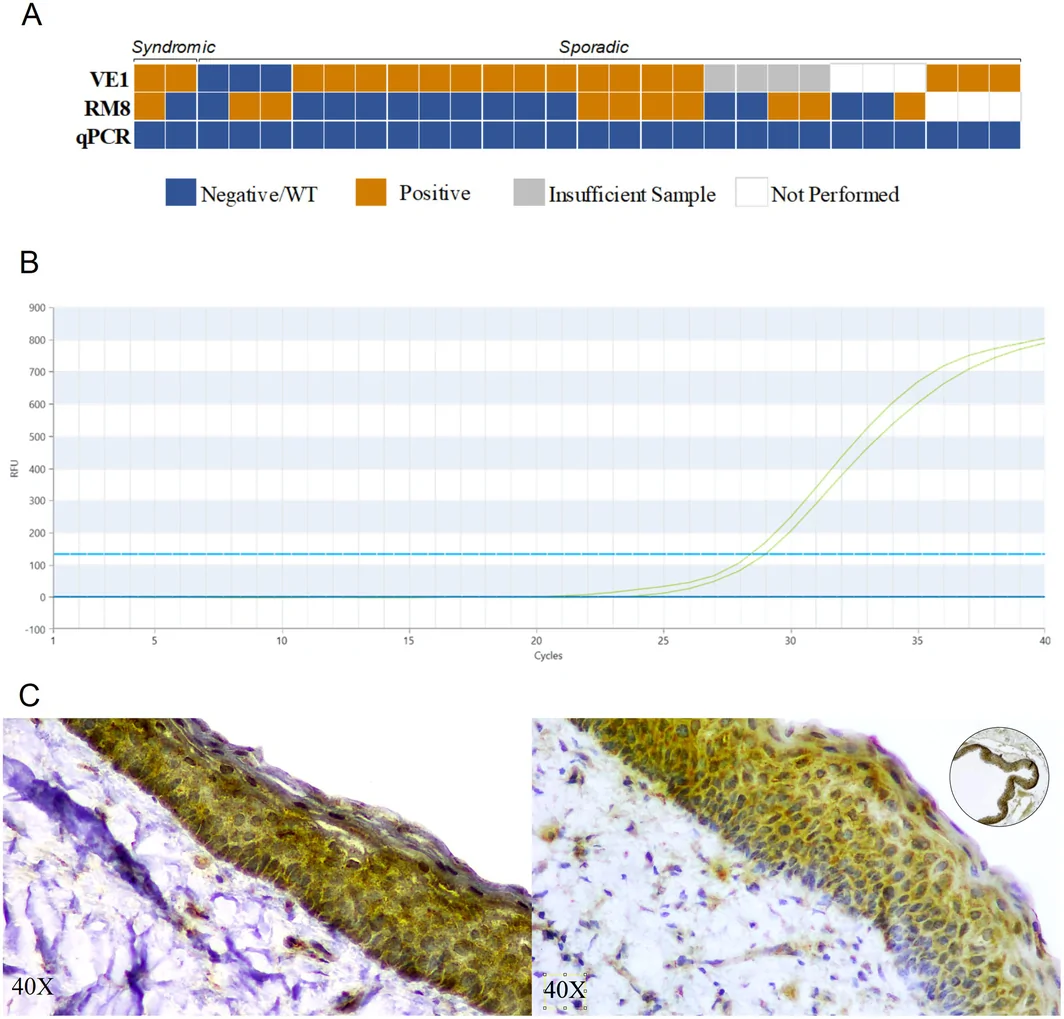
Immunohistochemical and molecular profile of *BRAF* p.V600E in odontogenic keratocysts. (A) Heatmap showing the distribution of immunohistochemical (IHC) staining for VE1 and RM8 monoclonal antibodies and qPCR results for *BRAF* among sporadic and syndromic OKCs. Each column represents an individual case. (B) Representative qPCR amplification curve demonstrating absence of *BRAF* amplification in all OKC samples, with amplification observed only in the positive controls (ameloblastoma harboring *BRAF* p.V600E). (C) Representative IHC micrographs of RM8 and VE1 positive‐staining in OKC samples.

No *BRAF* mutation was detected by qPCR, despite IHC positivity, indicating molecular‐immunophenotypic discordance. A systematic review by our group aligned with studies reporting absence of *BRAF* mutation (Cunha, J. da, et al. 2025). Among these studies, Cha et al. (2017) remains the only one to report a molecularly confirmed *BRAF* p.V600E mutation in OKC (Cha et al. 2017). However, subsequent studies reported negative results (França et al. 2018; Cunha, J. da, et al. 2025; Gomes et al. 2023; Togni et al. 2022). França et al. suggested that Cha's finding likely reflects contamination rather than a true mutation, a view supported by our data (França et al. 2018).

Discordance between IHC and qPCR is reported in colorectal and melanocytic tumors when VE1 is used (Krishnamurthy et al. 2018; Fisher et al. 2014). Although validated in melanomas and ameloblastomas (Martins‐de‐Barros et al. 2022; Fisher et al. 2014), VE1 may show nonspecific cytoplasmic reactivity in degraded tissue (Fisher et al. 2014), while RM8 often improves sensitivity but may vary with platform and antigen retrieval (Jain et al. 2020). TMA is a validated approach for immunohistochemical assessment; however, in cystic epithelial lesions such as OKC, sampling heterogeneity may influence apparent immunoreactivity, which may partly explain the higher positivity observed with VE1 compared with RM8.

The persistent absence of *BRAF* mutations supports that OKC pathogenesis is independent of MAPK activation. Conversely, *PTCH1* mutations and SHH pathway deregulation remain the most consistent alteration (Gomes et al. 2023). Thus, occasional IHC reactivity in *BRAF*–wild‐type OKCs likely reflects nonspecific antibody binding in keratin‐rich tissues (Ritterhouse and Barletta 2015). VE1 can show low‐level cross‐reactivity with non‐mutated BRAF fragments or cytoskeletal proteins that mimic the V600E epitope (Estrella et al. 2015; Ritterhouse and Barletta 2015), and RM8 may show similar background staining (Martins‐de‐Barros et al. 2022). Combined with epithelial fragility and TMA use, these factors may explain the false‐positive immunoreactivity despite negative molecular results.

When qPCR was used, RM8 showed 58.3% specificity (95% CI: 36.6–77.9), whereas VE1 showed only 19.0% (95% CI: 5.4–41.9); sensitivity was unassessable due to absence of molecular positives. These values contrast with the near‐perfect performance reported in ameloblastoma, where both sensitivity and specificity exceeded 95% and 100%, respectively (Martins‐de‐Barros et al. 2022). Direct comparison of the two antibodies in cases with paired results (*n* = 18) revealed only moderate concordance (*κ* = 0.37) and a significant discordance by McNemar's exact test (*p* = 0.031), driven by VE1‐positive/RM8‐negative discrepancies.

Collectively, these findings show that IHC is not a reliable indicator of *BRAF* mutational status in OKC. Positive immunoreactivity likely reflects false‐positive staining from off‐target binding or cross‐reactivity, a pattern noted for VE1 (França et al. 2018; Jain et al. 2020; Togni et al. 2022). Clinically, these false positives may prompt consideration of BRAF‐targeted therapies in lesions unlikely to benefit, exposing patients to unnecessary risks and costs without therapeutic gain.

In conclusion, no molecular evidence of *BRAF* p.V600E was detected in OKC, and VE1/RM8 immunoreactivity represented false‐positive staining. The low specificity observed for both antibodies, combined with the consistent absence across studies, demonstrates that BRAF IHC is unreliable for determining mutational status in OKC and should be interpreted with caution to avoid diagnostic or therapeutic misdirection. Although OKC is unlikely to involve MAPK pathway alterations, the presence of a wild‐type *BRAF* profile does not exclude the possibility of alternative oncogenic mechanisms beyond the scope of this analysis.

## Author Contributions

**Raisa Severino‐Lazo:** conceptualization, writing – review and editing, writing – original draft, investigation, data curation, formal analysis, methodology. **Jéssica da Silva Cunha:** investigation, resources. **Allan Vinícius Martins‐de‐Barros:** methodology, writing – review and editing. **Karina Helen Martins:** validation, visualization. **Adauto Gomes Barbosa Neto:** methodology, validation, formal analysis. **Jorge Esquiche León:** writing – review and editing, methodology. **Marianne de Vasconcelos Carvalho:** conceptualization, supervision, writing – review and editing.

## Funding

Jorge Esquiche León has received research grants (2016/11419‐0; 2022/07479‐9 and 2022/12760‐9) from State of São Paulo Research Foundation (FAPESP) and research grant (304241/2021‐0) from National Council for Scientific and Technological Development (CNPq). This work was supported by grants from the Coordination for the Improvement of Higher Education Personnel (CAPES, Coordenação de Aperfeiçoamento de Pessoal de Nível Superior).

## Ethics Statement

All procedures performed in studies involving human participants were in accordance with the ethical standards of the institutional and/or national research committee and with the 1964 Helsinki declaration and its later amendments or comparable ethical standards. As this study did not include research involving human participants, ethical approval was not required.

## Consent

The authors have nothing to report.

## Conflicts of Interest

The authors declare no conflicts of interest.

## Data Availability

The data that support the findings of this study are available from the corresponding author upon reasonable request.
